# The Evaluation of Antibiotic Consumption at the Inpatient Level in Kazakhstan from 2011 to 2018

**DOI:** 10.3390/antibiotics9020057

**Published:** 2020-02-02

**Authors:** Gulzira Zhussupova, Galina Skvirskaya, Vladimir Reshetnikov, Viktorija Dragojevic-Simic, Nemanja Rancic, Dinara Utepova, Mihajlo Jakovljevic

**Affiliations:** 1 The Republican State Enterprise on the Right of Economic Management “Republican Center for Health Development” of the Ministry of Health of the Republic of Kazakhstan, 010000 Nur-Sultan, Kazakhstan; utepova-88@mail.ru; 2 N.A. Semashko Department of Public Health and Healthcare I.M. Sechenov First Moscow State Medical University (Sechenov University), 119991 Moscow, Russia; gskvirskaya@mail.ru (G.S.); resh1960@mail.ru (V.R.); sidartagothama@gmail.com (M.J.); 3 The Centre for Clinical Pharmacology, Medical Faculty, Military Medical Academy, University of Defence, Crnotravska 17, 11002 Belgrade, Serbia; vdragsim@gmail.com (V.D.-S.); nece84@hotmail.com (N.R.); 4 Department of Global Health Economics and Policy, Faculty of Medical Sciences, University of Kragujevac, 34000 Kragujevac, Serbia; 5 Institute of Comparative Economics, Hosei University, 4342 Aihara-cho, Machida-shi, Tokyo 194-0298, Japan

**Keywords:** medicine consumption, antimicrobial medicines, inpatient level, Kazakhstan

## Abstract

Antimicrobial agents have a rather special position due to their importance as essential medicines for the treatment of infectious diseases. Evidence-based prescriptions are needed to optimize the use of antimicrobials in humans, as well as to decrease antimicrobial resistance. The aim of this study was to assess the inpatient consumption of antimicrobial drugs for systemic use in the period 2011–2018 in Kazakhstan. This article presents the results of an evaluation of the inpatient use of antibacterial drugs for systemic use (group J01) for the period 2011–2018 using the anatomical therapeutic chemical (ATC) classification)/defined daily dose (DDD) methodology recommended by the World Health Organization. Inpatient antimicrobial utilization is expressed as DDDs/1000 inhabitants/day (DID). The results of the assessment for inpatient antibiotic use (over an eight-year period) showed a decrease in the total consumption of antibiotics for systemic action in Kazakhstan (2011: 12.72 DID; 2018: 2.74 DID). Among oral formulations, levofloxacin was consumed the most, and cefazolin was consumed the most among the parenteral formulations of antimicrobials. The three drugs consumed the most included cefazolin (first-generation cephalosporin), ceftriaxone (third-generation cephalosporin), and cefuroxime (second-generation cephalosporin). The total consumption of antibacterials for systemic action in Kazakhstan decreased during the analyzed period, but there was an irrational use of certain groups of drugs.

## 1. Introduction

Antimicrobial agents have a rather special position due to their importance as essential medicines: they satisfy the priority healthcare needs of the population and are the foundation for treating the most infectious diseases [1,2].

The World Health Organization has identified key factors in antimicrobial resistance (AMR) development. Thus, AMR threatens the effective prevention and treatment of the ever-increasing number of infections caused by bacteria, parasites, viruses, and fungi [3]. The rise in antimicrobial resistance is a prominent public health concern because it has an impact on morbidity and the mortality of patients and is the cause of growing costs affecting healthcare systems [4,5]. Evidence-based prescriptions through effective, rapid, low-cost diagnostic tools are needed to optimize the use of antimicrobials in humans and animals [6,7].

In order to assess whether prescribing antibiotics in Kazakhstan is rational, an evaluation of the inpatient consumption of antimicrobial drugs for systemic use in the period 2011–2018 was conducted.

## 2. Results

The consumption of antimicrobials for systemic use at the inpatient level in the period 2011–2018 decreased constantly (starting with a sharp decline in use in 2014), amounting to 2.3 defined daily doses (DDDs)/1000 inhabitants/day (DID) compared to the year 2011, which was 12.7 DID.

**Figure 1:**
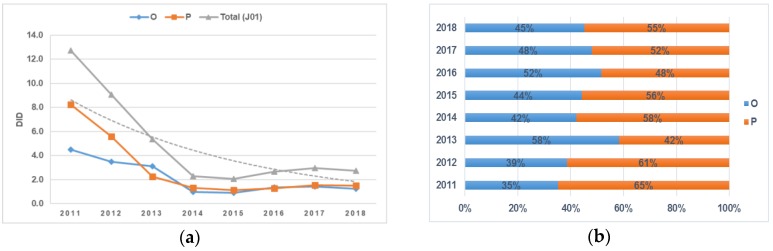
(**a**) Dynamics of changes in antimicrobial consumption according to the route of administration (oral: O; parenteral: P) in Kazakhstan in the period 2011–2018; (**b**) dynamics of changes in the proportion (%) of antimicrobial consumption according to the route of administration (oral: O; parenteral: P) in Kazakhstan in the period from 2011 to 2018.

The analysis of antibiotic consumption according to the route of administration also revealed a decrease in the consumption of both oral (O) and parenteral (P) forms of drug formulations from 2011 to 2018. Between 2011 and 2014, the consumption of parenteral antibiotics decreased dramatically, reaching 1.3 DID, and no further significant changes were observed. A relative increase (58% in comparison to parenteral formulations) was observed in oral antibiotic use between 2012 and 2013, amounting to 3.1 DID, but then it gradually decreased to 1.2 DID by 2018. The proportion of oral antibiotic consumption exceeded parenteral antibiotics only in 2013 and 2016, and in the other examined years, parenteral antibiotic consumption dominated: the difference was 30% in 2011, 22% in 2012, 16% in 2014, 4% in 2017, and 10% in 2018 (Figure 1a,b).

**Figure 2:**
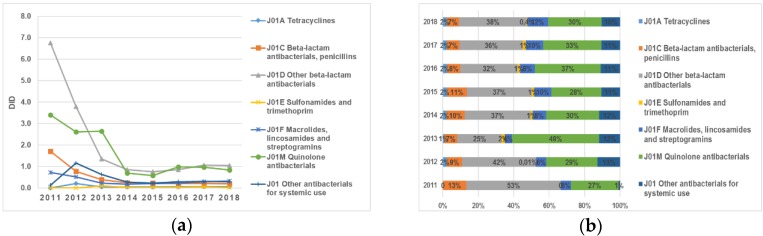
(**a**) Dynamics of changes in the most consumed pharmacological groups of antibiotics purchased within the context of guaranteed free medical care in the period 2011–2018; (**b**) dynamics of changes in the proportions of the most consumed pharmacological groups of antibiotics purchased in the context of guaranteed free medical care for the period 2011–2018 by pharmacological group.

The antibiotics belonging to the pharmacological groups “J01D Other beta-lactam antibacterials” and “J01M Quinolone antibacterials” remained the most consumed antibacterials for systemic use during the eight-year period (2011–2018) (Figure 2a,b). The largest changes in utilization were observed in the group “J01D Other beta-lactam antibacterial”, with a sharp decrease in DID from 6.75 DID in 2011 to 1.35 in 2013 DID: in 2018, it accounted for only 1.05 DID. At the same time, the proportion of high-generation cephalosporin and fluoroquinolone consumption throughout the whole analyzed period was relatively stable and always took the leading position (Figure 2a,b).

It should also be noted that the decline in the consumption of antibiotics belonging to the group “J01C Beta-lactam antibacterials, penicillins” was 76%: from 3.4 in 2011 to 0.83 in 2018 (Figure 2a,b).

**Figure 3:**
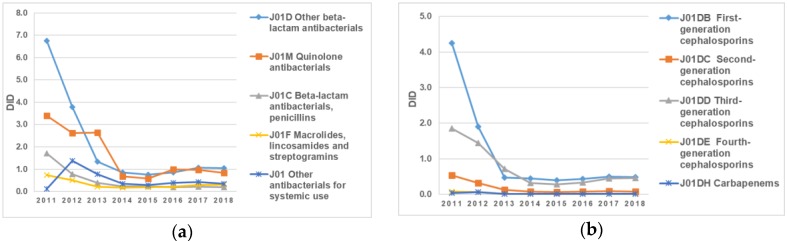
(**a**) Dynamics of changes in the most consumed pharmacological groups of antibiotics purchased in the context of guaranteed free medical care for the period 2011–2018 by pharmacological group; (**b**) dynamics of changes in antimicrobial consumption according to pharmacological group in Kazakhstan in the period 2011–2018.

The most frequently used antibiotics in 2017 were first- and third-generation cephalosporins, which accounted for 46% and 44%, respectively (Figure 3a). All consumption of first-generation cephalosporins in 2018 was related to cefazolin. In addition, a gradual decrease in carbapenem consumption (25% in comparison to 2011) to 0.01 DID was noticed in 2018 (Figure 3b).

Concerning parenteral forms of vancomycin, there was a 67% reduction in consumption in 2018 in comparison to 2011, amounting only to 0.01 DID.

**Figure 4:**
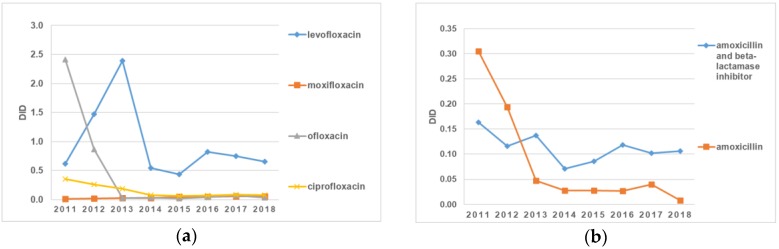
(**a**) The consumption of ofloxacin, ciprofloxacin, levofloxacin, and moxifloxacin in the Kazakh population between 2011 and 2018; (**b**) a comparative analysis between the consumption of amoxicillin and the consumption of amoxicillin with a beta-lactamase inhibitor.

The line graphs in Figure 4a present information concerning the consumption of fluoroquinolone antibiotics in the period from 2011 to 2018. As can be seen, there were significant fluctuations in the use of levofloxacin compared to other antibiotics in this group throughout the entire period. This increased from 0.62 DID to 2.39 DID from 2011 to 2013, followed by a dramatic reduction in value to 0.44 DID in 2015. However, levofloxacin consumption grew after 2015 and reached almost 0.82 DID in 2016. In contrast, the consumption of moxifloxacin and ciprofloxacin remained stable over the eight years. Regarding ofloxacin, there was a sharp drop in the consumption of this drug between 2013 and 2014, from 2.39 DID to 0.54 DID. After that, there were no significant changes until the end of the study period (Figure 4a).

A comparative analysis between the consumption of amoxicillin and amoxicillin with a beta-lactamase inhibitor showed a decrease in the latter of 35% in 2018 in comparison to 2011, while the proportion of amoxicillin consumption fell steadily by 89% between 2011 and 2018 (Figure 4). Just between 2013 and 2014, the DID of amoxicillin with a beta-lactamase inhibitor decreased dramatically from 0.14 to 0.07. Over the next two years (2014–2016), there was a significant rise to 0.12 DID. On the other hand, there was a downward trend in the consumption of amoxicillin over the entire period of time, with an increase of 0.04 DID in 2017. In general, it can be seen that the consumption of amoxicillin with a beta-lactamase inhibitor was higher than the consumption of amoxicillin itself during practically the entire examined period (Figure 4b).

**Figure 5:**
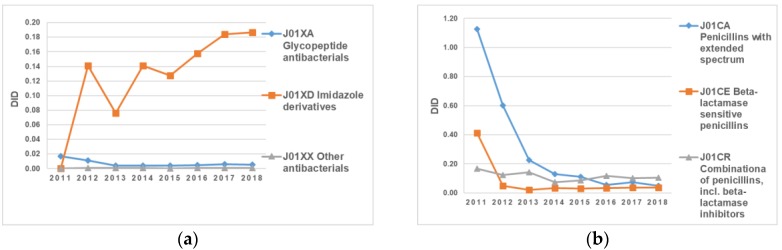
(**a**) Utilization of “J01XA Glycopeptide antibacterials”, “J01XD Imidazole derivates”, and “J01XX Other antibacterials” in Kazakhstan from 2011 to 2018; (**b**) utilization of “J01CA Penicillins with extensions”, J01CE Beta-lactamase-sensitive penicillins”, and “J01CR Combinations of penicillins” in Kazakhstan from 2011 to 2018.

Figure 5a illustrates data about consumption of the following groups: “J01XA Glycopeptide antibacterials”, “J01XD Imidazole derivates”, and “J01XX other antibacterials” over the eight-year period. The use of J01XD imidazole derivates fluctuated substantially in comparison to the other two groups of antimicrobials, which remained unchanged and followed a similar pattern over the entire period. The intake of J01XD Imidazole derivates dropped markedly from 0.14 DID to 0.08 DID in 2013 alone. In 2014, consumption increased back to 0.14 DID. In addition to that, it should be noted that the consumption of J01XD Imidazole derivates experienced an increase from 2015 onward (Figure 5a).

The use of J01СA Penicillin with an extended spectrum tended to decline until 2016 in comparison to the J01CE and J01CR groups, which mostly remained unchanged and followed a similar pattern over the entire period (Figure 5b).

The analysis for 2018 noted that among the top 10 most consumed antibacterial drugs, which are presented in Table 1, levofloxacin had the leading position, accounting for 50% of the total oral intake of antibacterials. Cefuroxime, ofloxacin, and amoxicillin proved to be the least consumed antibacterial drugs, and together they accounted for just 3% of the total oral antibacterial intake. The situation was similar in 2017. The consumption rate of levofloxacin was 0.72 DID, which accounted for 50% of the total consumption of oral antibacterials (Table 1).

In the analysis of the consumption of parenteral antibacterial drugs in 2018, the leader in terms of consumption was cefazolin, whose DID value was 0.48, which accounted for 32% of the total consumption of parenteral forms of antibiotics (followed by ceftriaxone (DID value of 0.38) and metronidazole (DID value of 0.19)) (Table 2). Ceftazidime, benzylpenicillin, and cefotaxime were the least consumed. In 2017, cefazolin, ceftriaxone, and metronidazole were also the most consumed drugs, with cefazolin consumption being 50% of the total consumption of parenteral antibacterial drugs. Ciprofloxacin, whose DID value was 0.03, was the least consumed parenteral antibiotic (Table 2).

Detailed data on oral and parenteral antibacterial use according to the 10 most consumed drugs in 2017 and 2018 are shown in Table 1 and Table 2.

In Table 3, our results are expressed in the context of WHO quality indicators on the use of antimicrobials (Table 3).

## 3. Discussion

The total consumption of antibacterials for systemic use in Kazakhstan decreased by five times the original amount after 2011. In 2009, Kazakhstan began to implement reforms in the health sector. As part of that reform, drug information centers were established in all regions of the country to improve drug literacy among the population and among medical workers. In addition, since 2015, Kazakhstan has actively participated in “World Antibiotic Awareness Week”, which was initiated by the World Health Organization. It is very likely that the above measures have influenced the positive dynamics of antibiotic consumption.

The most important result in terms of the literature is that antibacterial consumption in Kazakhstan (on an inpatient level) throughout the entire study was a maximum of 12.7 DID (2011). This is low compared to other countries (because coverage was at the stationary level). In Eastern Europe, total consumption, including at the outpatient level, ranged from 15.3 DID (Armenia) to 42.3 DID (Turkey) in 2011. However, according to another Kazakhstan study with commercial data, total antibiotic consumption at the outpatient and inpatient level in 2011 was an average of 23.6 DID [8].

In reviewing recent drug utilization studies in Kazakhstan, we found a number of them to be comparable to our results. Studies in Kazakhstan on antibiotic consumption using the anatomical therapeutic chemical (ATC) classification/DDD methodology recommended by the WHO were reviewed. However, these studies used data on antibiotic consumption for different levels and types of medical care. For example, one study of antimicrobial medicine consumption in Eastern Europe and Central Asia, which described the results of a comparative analysis and included Kazakhstan, was based on a commercial consumption data source that provided coverage for around 80–85% of hospital and community sales in Kazakhstan [8]. Therefore, a full comparison of our research data to the above data is not relevant because our research was conducted only at a stationary level. In addition, a recent study on the appropriate use of antibiotics in the Republic of Srpska was conducted, taking into account data at the outpatient level [9]. This is also not comparable to data on the use of antibiotics at the inpatient level in Kazakhstan. One WHO study on antibiotic use in Eastern Europe overlapped with the results of cross-national database research that was conducted in coordination with the WHO Regional Office for Europe. The methods and materials used in this study were based on wholesale trade information [10]. The results of this study were more or less comparable to our survey, as the “Single Distributor” in Kazakhstan is a wholesale buyer of medicines.

Another important result was discovering that parenteral antibiotics were the most commonly used antibiotics, probably because the study was done at the stationary level. Parenteral antibiotic treatment is a common practice, accounting for 46.4% of total antibiotic intake in Azerbaijan (mainly ampicillin; 5.3 DID) and 31.1% of total antibiotic intake in Tajikistan (mainly ceftriaxone; 4.7 DID) [10]. In both studies (in Kazakhstan and in Eastern Europe), there was the same tendency for first- and third-generation cephalosporins to be the most commonly consumed (between 48% for inpatient use and 90% for inpatient and outpatient use), particularly ceftriaxone. The three most commonly used oral antibacterials (levofloxacin, azithromycin, and clarithromycin) all have very high oral bioavailability. Parenteral or oral switching of these agents is very common in hospitals to reduce the unnecessary use of parenteral antibiotics in relatively clinically stable patients. To some extent these prescription patterns were comparable to the recent case of antibiotic resistance noted in Syria [11].

A third important result was in the analysis of pharmacological groups of antibacterials and certain antibiotics according to WHO indicators. A comparative analysis of the consumption of the drugs of the group “J01M Quinolone antibacterials” revealed a steady leading position of the drug levofloxacin over the eight-year period. At the same time, according to the commercial data of another Kazakhstan study, quinolones were the third most consumed antibiotic group, representing from 7.5% to 24.6% of total J01 consumption [8]. Third place was occupied by the group “J01M Quinolone antibacterials” in European Union and European Economic Area (EU/EEA) countries (according to the surveillance report “Antimicrobial Consumption in the EU/EEA”) [12]. Concerning the group “J01F Macrolides, lincosamides and streptogramins” on an inpatient level in Kazakhstan, a sustainable reduction in consumption was noted (accounting for 56% and reaching its minimum in 2018 (0.36 DID compared to 0.73 DID in 2011)). When comparing this to WHO data, Montenegro and Serbia were the highest consumers of macrolides (15.8% and 19.5% of total antibiotic consumption, respectively), mainly azithromycin [10]. An analysis of the consumption of amoxicillin and amoxicillin with a beta-lactamase inhibitor (clavulanic acid or sulbactam) showed a four-fold decrease in the consumption of amoxicillin alone in 2018 in Kazakhstan at the inpatient level. There was a tendency toward increased consumption of combinations of penicillins in the countries in Eastern Europe (Georgia and Turkey) [10]. Thus, the assessment of antibacterial consumption by pharmacological group and drug revealed a similar pattern of consumption to that in Eastern Europe.

Currently, there is no guidance on the use of antibiotics in healthcare practices in Kazakhstan. Each medical organization usually develops its own guidelines, taking into account antibiotic sensitivities at the local level. The guidelines are based on the World Health Organization’s guidelines on the use of antimicrobials. At the same time, there is a practice of developing clinical protocols by professional associations in the country for certain nosologies, including the use of antibiotics for infectious diseases. In addition, in order to provide free medicine within Kazakhstan’s guaranteed free medical care, there are approved lists (Kazakhstan National Formulary, a list of medicines to be purchased by the “Single Distributor”, a list of orphan drugs). Probably, the regular assessment of antibiotic consumption using the ATC/DDD methodology will contribute to implementing national guidelines on the use of antibiotics.

In summary, the evaluation of the inpatient use of antibacterials for systemic use, taking into account WHO quality indicators on the use of antimicrobials, showed a positive trend in total consumption compared to other countries. However, the structure of consumption of antibacterials was similar to that of other countries, indicating a possible risk of the irrational use of specific groups of antibiotics or individual drugs.

There were some limitations to the study. New ATC codes and DDDs were included in the January 2019 version of the “ATC/DDD Index”. Changes to DDDs were implemented for several commonly used antibiotics (amoxicillin, amoxicillin with a beta-lactamase inhibitor, meropenem, colistin, cefepime, ampicillin, and ciprofloxacin). However, since our data were for a period before 2019, in our study the expenditure of antibiotics was calculated based on the old DDD values.

## 4. Materials and Methods

Data on the medicines purchased by the “Single Distributor of Kazakhstan” within the framework of guaranteed free medical care at the inpatient level for the period from 2011 to 2018 were used in this work.

The evaluation of antimicrobial use at the inpatient level was conducted using ATC (anatomical therapeutic chemical classification)/DDD (defined daily dose) methodology, which is internationally accepted for measuring medicine utilization within and across populations. The DDD is the amount of drug most commonly used in adults for the most common indication, which is very useful as a measure to describe and compare drug utilization patterns between countries and healthcare facilities. As far as data on inpatient antimicrobial utilization are concerned, they are expressed in DDDs/1000 inhabitants/day (DID) [13]. Data on the consumption of antibiotics were provided by the subordinate organization of the Ministry of Health of the Republic of Kazakhstan, the Single Distributor of Medicines, which carries out the purchase of medicines to provide a guaranteed amount of free medical care. The list of medicines purchased for this guaranteed free medical care is limited within the state budget. Therefore, the data on antibiotic consumption were reliable, but did not cover retail consumption. Statistical data such as population size, number of days per year, and average daily dose equal to the dose unit were also used to calculate inpatient antibiotic consumption.

The calculation of antibiotic consumption was done for the following reasons:

(1) A more detailed study on the consumption structure of antibacterial drugs for systemic action (with ATC code J01 for pharmacological subgroups);
(2) An analysis of the consumption of oral and parenteral antibacterials in terms of the most consumed top 10 drugs for 2017–2018;
(3) A determination of the main trends of antibacterial consumption at the stationary level in general.

In order to study the relationship between the proportions of consumption of high-generation cephalosporins and fluoroquinolones and the total consumption of antibiotics, statistical studies were conducted comparing the trends in total consumption of antibiotics to differences in the proportions of consumption of the individual groups of antibiotics.

To determine the DID of an indicator, calculations were made using Equations (1) and (2):

(1) $$\mathrm{DID}=\frac{\mathrm{DDDs}\times 1000}{\mathrm{population}\;\times \;365(\mathrm{or}\;366)\;(\mathrm{number}\;\mathrm{of}\;\mathrm{days}\;\mathrm{per}\;\mathrm{year})}$$

where DDDs represents the total number of DDDs, and

(2) $$\mathrm{DDDs}=\frac{\mathrm{UD}\times N}{\mathrm{DDD}}$$

where UD is unit dose in mg, N is the number of medicines, and DDD is the defined daily dose.

## 5. Conclusions

The results of the study showed that the total consumption of antibacterials for systemic use at the stationary level in Kazakhstan over an eight-year period steadily declined.

Throughout the entire study period, parenteral antibiotics were used more often than were oral antibiotics. The high consumption of parenteral antibiotics was due to the fact that the evaluation was conducted at the hospital level.

The study of the top 10 oral forms of antibacterials in Kazakhstan at the inpatient level showed that levofloxacin steadily remained the most consumed, making up almost half of all oral antibiotics. On the other hand, among the parenteral formulations of antimicrobials, cefazolin and ceftriaxone were the most often used, together comprising more than half of all parenteral forms of antibacterials (32% and 25%, respectively).

The most widely consumed antibiotics were the drugs of the J01D pharmacological group, which was represented mainly by cephalosporins and accounted for 32–53% of all antibiotics for systemic use. The three drug consumption leaders included cefazolin (first-generation cephalosporin), ceftriaxone (third-generation cephalosporin), and cefuroxime (second-generation cephalosporin). The overuse of cephalosporin causes alarming signals, and thus its use needs to be rational. Regarding the comparison of the consumption of amoxicillin to amoxicillin and a beta-lactamase inhibitor together, it should be noted here that the latter was used more often than amoxicillin alone, indicating an increase in bacterial resistance to amoxicillin itself.

The total utilization of fluoroquinolone was very high, from 26% to 49%. This is also very worrying data when recent information concerning the safety of these drugs are taken into account. 

Thus, the results of the assessment of inpatient antibiotic use over an eight-year period showed that despite a decrease in the total consumption of antibacterials for systemic action in Kazakhstan, there was an irrational use of certain groups of drugs. The results of this study should be used in the development of national guidelines for the appropriate use of antibiotics. The regular assessment of antibiotic consumption using the ATC/DDD methodology allows for the monitoring of antibiotic use and the effective control of antimicrobial resistance.

## Figures and Tables

**Table 1:** Comparative analysis of the consumption of the top 10 oral antibiotics for the period 2017–2018.

Oral INN *, 2017	DID	%	Oral INN *, 2018	DID	%
levofloxacin	0.72	50	levofloxacin	0.62	50
clarithromycin	0.16	12	azithromycin	0.16	13
azithromycin	0.11	8	clarithromycin	0.15	12
amoxicillin and clavulanic acid	0.09	6	amoxicillin and clavulanic acid	0.09	7
doxycycline	0.07	5	doxycycline	0.06	5
moxifloxacin	0.06	4	moxifloxacin	0.06	5
ofloxacin	0.05	4	ciprofloxacin	0.05	4
ciprofloxacin	0.05	4	cefuroxime	0.02	1
amoxicillin	0.04	3	ofloxacin	0.02	1
sulfamethoxazole and trimethoprim	0.04	3	amoxicillin	0.01	1
Total DID of oral antibiotics	1.42	100	Total DID of oral antibiotics	1.24	100

* INN: international nonproprietary name.

**Table 2:** Comparative analysis of the consumption of the top 10 parenteral antibiotics for the period 2017–2018.

Parenteral INN *, 2017	DID	%	Parenteral INN *, 2018	DID	%
cefazolin	0.50	32	cefazolin	0.48	32
ceftriaxone	0.36	24	ceftriaxone	0.38	25
metronidazole	0.18	12	metronidazole	0.19	12
amikacin	0.10	6	amikacin	0.07	5
cefuroxime	0.07	4	cefuroxime	0.07	4
ceftazidime	0.04	2	ampicillin	0.04	3
levofloxacin	0.04	2	levofloxacin	0.04	3
benzylpenicillin	0.04	2	ceftazidime	0.04	2
cefotaxime	0.04	2	benzylpenicillin	0.04	2
ciprofloxacin	0.03	2	cefotaxime	0.04	2
Total DID of parenteral antibiotics	1.54	100	Total DID of parenteral antibiotics	1.50	100

* INN: international nonproprietary name.

**Table 3:** WHO quality indicators on the use of antimicrobials. DID: defined daily dose (DDD)/1000 inhabitants/day.

WHO Indicators	2011	2012	2013	2014	2015	2016	2017	2018
Total utilization of antibiotics (J01) (DID)	12.72	9.07	5.37	2.30	2.06	2.67	2.96	2.74
Total utilization of beta-lactam antibiotics (penicillins (J01C) and cephalosporins (J01D)) as a % of total antibiotic use	66.49	50.34	32.34	47.50	47.74	39.98	43.03	45.13
Total utilization of penicillins (J01C) as a % of total antibiotic use	13.41	8.53	7.21	10.31	11.07	7.69	7.13	6.98
Utilization of combination penicillins (co-amoxiclav (J01CR02)) as a % of total antibiotic use	1.29	1.28	2.56	3.09	4.17	4.45	3.44	3.87
Total utilization of cephalosporins (J01D) (DID)	6.75	3.79	1.35	0.86	0.76	0.86	1.06	1.05
Utilization of third- and fourth-generation cephalosporins (J01DD and J01DE) as a % of total antibiotic use	15.16	16.56	13.61	14.04	13.71	12.59	15.50	17.10
Utilization of third- and fourth-generation cephalosporins as a % of total cephalosporin use	28.56	39.60	54.17	37.76	37.40	39.00	43.17	44.83
Total utilization of macrolides, lincosamides, and streptogramins (J01F) (DID)	0.73	0.51	0.22	0.17	0.20	0.22	0.29	0.32
Total utilization of erythromycin (J01FA01), clarithromycin (J01FA09), and azithromycin (J01FA10) as a % of total antibiotic use	5.12	4.80	3.90	7.14	9.48	7.97	9.28	11.14
Total utilization of erythromycin as a % of total macrolide use, with a corresponding increase in clarithromycin and azithromycin	-	-	-	-	-	-	-	-
Total utilization of quinolones (J01M) (DID)	3.40	2.62	2.64	0.69	0.58	0.99	0.97	0.83
Total utilization of fluoroquinolones (J01MA) as a % of total antibiotic utilization	26.76	28.87	49.11	29.98	28.16	37.14	32.88	30.38
